# Whiskey Drinking

**Published:** 1875-01

**Authors:** 


### WHISKEY DRINKING.

Take any equal number of intemperate and temperate persons from fifteen to twenty years of age, while eighteen of the intemperate die, only ten die who are temperate; but between the ages of twenty and thirty, five intemperate persons die and only one temperate; between thirty and forty the proportion is four to one.

But look at it in another light. If a man is temperate up to twenty his chances for life is forty-two years, that is, he is likely to live until he is forty-four years old; if intemperate, he will most probably die at thirty-five; thus those who drink liquor freely while young, scarcely live out half their days, as a rule; living longer the less they "indulge." Then there is the cost of liquor-drinking. Mr. Ruffner, the very able and industrious Superintendent of Public Instruction in Virginia, says that the retail liquor stores in that State sell over eleven millions of dollars worth of liquor in a year, which is just about equal to the whole amount of wheat raised on its soil; and if you take into account what is sold in the shape of "bitters," which is really only liquor disguised, but more costly, a million or two, or more, is added to the amount, to say nothing of the loss of time while under the influence of liquor, and the expense to the State of prosecuting for crimes committed by the intemperate; and in addition the caring for individuals and families impoverished directly by the intemperate habits of some members of the family; and then there is the mortification and heart-agony of disgraced wives and sons and daughters. And yet our representative men in the halls of legislation, in almost every State, and in England, too, will give a man the privilege of selling liquor to any one who can pay the money, for ten or fifteen dollars a year, and this, too, in the very dawn of the twentieth century of Christian civilization.

———

In these days the contributions to medical resources are constant; now it is a Pill, an awful Pill, and now it is a pensive powder. The latest additions to the doctor's store are bones and banjo. A young Philadelphia girl who had, since childhood, been prevented from walking by a nervous spine, suddenly manifested, two years ago, a frantic liking for negro minstrel entertainments. Physicians long had been in vain, and the wretched maiden in despair turned to the weird and mournful beauty of these performances for distraction. Every evening for those two years she listened to the bounding freshness of the jokes, the soft pathos of the bones, and as time wore on, grew stronger and stronger. She is now perfectly well, and her friends attribute this pleasant change altogether to the minstrelsy.

